# Supplementary material for: Ivermectin Effect on In-Hospital Mortality and Need for Respiratory Support in COVID-19 Pneumonia: Propensity Score-Matched Retrospective Study
Source: Viruses. 2023 May 10;15(5):1138. doi: 10.3390/v15051138 (PMC10222278; doi:10.3390/v15051138)
Supplement: Supplementary file 1 [file viruses-15-01138-s001.zip › viruses-2392284-supplementary.pdf]

Supplementary tables

Supplementary Table S1. **Logistic regression analysis for need of ICU admission.**

	Univariable			Multivariable		
	OR	95% CI	P value	aOR	95% CI	P value
Group (IVM vs control)	0.91	0.49, 1.68	0.75	1.04	0.51, 2.11	0.92
Age	0.99	0.97, 1.01	0.18			0.54
Sex (female vs male)	0.78	0.41, 1.48	0.45			0.29
Country of birth (non-European vs European)	1.09	0.59, 2.03	0.78			0.59
Pandemic wave (2nd vs 1st)	0.83	0.17, 3.99	0.82			0.19
Pandemic wave (3rd vs 1st)	0.41	0.09, 1.94	0.26			0.23
SEIMC score †	0.96	0.91, 1.02	0.17			
Days since symptoms onset	0.99	0.92, 1.08	0.92			
Arterial hypertension	1.20	0.64, 2.23	0.58			
Diabetes mellitus	1.84	0.89, 3.82	0.10			
Hypercholesterolemia	0.67	0.33, 1.36	0.27			
Obesity	1.24	0.60, 2.55	0.57			
Smoking	1.16	0.28, 4.82	0.83			
COPD	0.44	0.09, 2.09	0.30			
Asthma	0.65	0.13, 3.22	0.60			
Chronic kidney disease	0.15	0.02, 1.17	0.07			
Cerebrovascular disease	0.37	0.04, 3.18	0.37			
Neoplasia	0.42	0.14, 1.30	0.13			
Inflammatory bowel disease	0.68	0.18, 2.55	0.56			
ICU candidate (no vs yes)	0.12	0.02, 0.90	0.04*			
ACE inhibitors	1.79	0.68, 4.71	0.24			
ARA-II	1.21	0.58, 2.50	0.62			
Inhaled corticosteroids	0.64	0.20, 2.02	0.44			
Inhaled beta-agonists	0.61	0.16, 2.27	0.46			
Inhaled anticholinergics	0.56	0.12, 2.73	0.48			
COVID-19 diagnostic technique (antigen vs PCR)	1.03	0.48, 2.22	0.93			
Infiltrate at X-ray (unilateral vs bilateral)	0.13	0.02, 0.99	0.05*			0.09
Systolic blood pressure (mmHg)	1.01	0.99, 1.03	0.26			
Diastolic blood pressure (mmHg)	0.98	0.95, 1.01	0.15			
Heart rate (beats per minute)	1.00	0.98, 1.02	0.89			
Temperature (°C)	1.35	1.01, 1.81	0.05*			
O_2 saturation (%)	0.95	0.91, 1.00	0.07			0.47
Respiratory rate (breaths per minute)	1.05	0.95, 1.16	0.38			
Glucose (mg/dL)	1.00	0.99, 1.01	0.75			
Urea (mg/dL)	1.00	0.99, 1.01	0.96			
Creatinine (mg/dL)	0.97	0.52, 1.79	0.91			
eGFR (MDRD-4 method) (mL/min/1.73m2)	1.00	0.99, 1.01	0.97			
Bilirubin (mg/dL)	1.02	0.42, 2.52	0.96			

GOT (UI/L)	1.02	1.00, 1.05	0.05			
GGT (UI/L)	1.00	1.00, 1.01	0.05			0.41
LDH (UI/L)	1.00	0.99, 1.01	0.19			
Creatinine-kinase (UI/L)	1.00	0.99, 1.00	0.35			
Sodium (mmol/L)	1.01	0.97, 1.07	0.59			
Potassium (mmol/L)	0.34	0.16, 0.72	0.005**	0.42	0.18, 0.99	0.05*
Ferritin (µg/L)	1.00	1.00, 1.00	0.46			
C-reactive protein (mg/dL)	1.08	1.04, 1.13	<0.001***	1.09	1.03, 1.14	0.002**
Procalcitonin (ng/mL)	4.65	1.39, 15.5	0.01*			0.26
Troponin I (pg/mL)	0.98	0.96, 1.01	0.29			
Hemoglobin (g/L)	1.00	0.99, 1.02	0.75			
Leucocytes (10^9 cel/L)	1.09	0.99, 1.21	0.08			0.93
Lymphocytes (10^9 cel/L)	1.16	0.89, 1.51	0.28			
Neutrophils (10^9 cel/L)	1.02	0.99, 1.05	0.22			
Platelets (10^9 cel/L)	1.00	0.99, 1.00	0.89			
aPTT (s)	1.03	0.98, 1.07	0.23			
D-dimer (mg/dL)	1.18	0.83, 1.69	0.36			
Arterial pH	1.02	0.97, 1.07	0.49			
Arterial pO_2 (mmHg)	1.01	0.99, 1.02	0.39			
Arterial pCO_2 (mmHg)	0.96	0.88, 1.05	0.38			
Tocilizumab	3.24	0.70, 14.9	0.13			0.21
Remdesivir	1.12	0.59, 2.14	0.73			0.16
Corticosteroids	NC	NC	NC			

*p<0.05; **p<0.01; ***p<0.001

ACE; angiotensin converting enzyme, aOR, adjusted odds ratio; aOR: adjusted odds ratio; aPTT, activated partial thromboplastin time; ARA-II: angiotensin II antagonist receptors; CI, confidence interval; COPD: chronic obstructive pulmonary disease, ICU, intensive care unit; LDH, lactate dehydrogenase; OR, odds ratio PCR: polymerase chain reaction; SEIMC: Spanish Society of Infectious Diseases and Clinical Microbiology; NC: not calculable (all but 1 patient received corticosteroids)

† SEIMC score is a prediction score based on age, age-adjusted low saturation of oxygen, neutrophil-to-lymphocyte ratio, estimated glomerular filtration rate, dyspnoea and sex. It has proven to be a useful tool to predict 30-day mortality probability among hospitalised patients with COVID-19

Hosmer-Lemeshow test, p=0.96, Chi^2 p= 0.002, Percentage correct classification 71.3%, R^2: 0.130

Supplementary Table S2. **Linear regression analysis for length of hospital stay.**

	Univariable			Multivariable		
	β	95% CI	P value	aβ	95% CI	p-value
Group (IVM vs control)	−0.09	−1.90, 1.72	0.93	0.31	−1.31, 1.94	0.70
Age	0.06	0.00, 0.11	0.04*			0.67
Sex (female vs male)	−1.19	−3.04, 0.65	0.20			0.90
Country of birth (non-European vs European)	−1.59	−3.40, 0.21	0.08			0.72
Pandemic wave (2nd vs 1st)	−3.53	−9.43, 2.37	0.24			
Pandemic wave (3rd vs 1st)	−4.47	−10.30, 1.38	0.13			
SEIMC score †	0.24	0.08, 0.40	0.004**			
Days since symptoms onset	−0.15	−0.39, 0.08	0.20			
Arterial hypertension	1.24	−0.62, 3.11	0.19			
Diabetes mellitus	1.79	−0.59, 4.17	0.14			
Hypercholesterolemia	1.04	−0.95, 3.03	0.30			
Obesity	0.97	−1.30, 3.25	0.40			
Smoking	1.83	−2.35, 5.99	0.39			
COPD	3.22	−0.70, 7.14	0.11			
Asthma	1.95	−2.21, 6.12	0.36			
Chronic kidney disease	2.75	−1.18, 6.67	0.17			
Cerebrovascular disease	1.71	−3.07 −6.50	0.48			
Neoplasia	0.98	−1.89, 3.86	0.50			
Inflammatory bowel disease	1.88	−1.70, 5.47	0.30			
ICU candidate (no vs yes)	2.61	−1.01, 6.23	0.16			
ACE inhibitors	1.78	−1.33, 4.89	0.26			
ARA-II	−0.22	−2.49, 2.06	0.85			
Inhaled corticosteroids	2.51	−0.58, 5.61	0.11			
Inhaled beta-agonists	0.25	−3.21, 3.71	0.89			
Inhaled anticholinergics	3.00	−1.15, 7.16	0.16			
COVID-19 diagnostic technique (antigen vs PCR)	−2.65	−4.97, −0.32	0.03*	−2.54	−4.59, 0.49	0.02*
Infiltrate at X-ray (unilateral vs bilateral)	−2.10	−5.13, 0.94	0.17			
Systolic blood pressure (mmHg)	0.03	−0.03, 0.08	0.32			
Diastolic blood pressure (mmHg)	−0.04	−0.12, 0.05	0.40			
Heart rate (beats per minute)	0.04	−0.01, 0.09	0.10			
Temperature (°C)	0.67	−0.18, 1.53	0.12			
O_2 saturation (%)	−0.52	−0.73, −0.30	<0.001***	−0.28	−0.53, 0.03	0.03*
Respiratory rate (breaths per minute)	0.06	−0.25, 0.37	0.69			
Glucose (mg/dL)	0.01	−0.01, 0.02	0.60			
Urea (mg/dL)	0.06	0.01, 0.10	0.01*			
Creatinine (mg/dL)	3.39	1.05, 5.74	0.005**			
eGFR (MDRD-4 method) (mL/min/1.73m2)	−0.05	−0.08, −0.01	0.007**	−0.03	−0.06, 0.01	0.04*
Bilirubin (mg/dL)	−0.16	−3.20, 2.89	0.92			
GOT (UI/L)	0.01	−0.06, 0.08	0.73			
GGT (UI/L)	−0.01	−0.01, 0.01	0.86			
LDH (UI/L)	0.01	−0.01, 0.02	0.30			
Creatinine-kinase (UI/L)	0.01	0.00, 0.02	0.02*			

Sodium (mmol/L)	0.01	−0.07, 0.09	0.78			
Potassium (mmol/L)	0.32	−1.42, 2.06	0.72			
Ferritin (µg/L)	0.00	−0.01, 0.01	0.77			
C-reactive protein (mg/dL)	0.18	0.05, 0.31	0.007**			0.65
Procalcitonin (ng/mL)	5.71	2.08, 9.34	0.002**	4.18	0.82, 7.54	0.02*
Troponin I (pg/mL)	0.08	0.00, 0.16	0.04*			0.86
Hemoglobin (g/L)	−0.01	−0.06, 0.03	0.56			
Leucocytes (10^9 cel/L)	0.17	−0.13, 0.47	0.26			
Lymphocytes (10^9 cel/L)	0.56	0.14, 0.98	0.01*	0.58	0.21, 0.95	0.003**
Neutrophils (10^9 cel/L)	0.07	−0.01, 0.16	0.084			0.80
Platelets (10^9 cel/L)	−0.01	−0.02, −0.00	0.030*	−0.010	−0.02, −0.002	0.02*
aPTT (s)	0.12	−0.05, 0.29	0.16			
D-dimer (mg/dL)	0.80	−0.41, 2.01	0.19			
Arterial pH	−7.30	−39.4, 24.8	0.65			
Arterial pO_2 (mmHg)	−0.01	−0.06, 0.05	0.84			
Arterial pCO_2 (mmHg)	−0.23	−0.49, 0.03	0.086			
Tocilizumab	7.62	1.02, 14.30	0.02*	8.81	3.05, 14.60	0.003**
Remdesivir	1.88	0.01, 3.75	0.05*			0.92
Corticosteroids	NC	NC	NC			

*p<0.05; **p<0.01; ***p<0.001

ACE; angiotensin converting enzyme, aOR, adjusted odds ratio; aPTT, activated partial thromboplastin time; ARA-II: angiotensin II antagonist receptors; CI, confidence interval; COPD: chronic obstructive pulmonary disease, ICU, intensive care unit; LDH, lactate dehydrogenase; OR, odds ratio PCR: polymerase chain reaction; SEIMC: Spanish Society of Infectious Diseases and Clinical Microbiology; NC: not calculable (all but 1 patient received corticosteroids)

† SEIMC score is a prediction score based on age, age-adjusted low saturation of oxygen, neutrophil-to-lymphocyte ratio, estimated glomerular filtration rate, dyspnoea and sex. It has proven to be a useful tool to predict 30-day mortality probability among hospitalised patients with COVID-19

ANOVA F p-value: <0.001, R2: 0.265, Durbin-Watson: 1.95
